# Editorial: Advances in Shannon-based communications and computations approaches to understanding information processing in the brain

**DOI:** 10.3389/fncom.2023.1352772

**Published:** 2024-01-04

**Authors:** James Tee, Giorgio M. Vitetta

**Affiliations:** ^1^Department of Electrical and Computer Engineering, University of Canterbury, Christchurch, New Zealand; ^2^Department of Engineering “Enzo Ferrari”, University of Modena and Reggio Emilia, Modena, Italy

**Keywords:** Shannon, information processing, brain, communication, computation, channel capacity, modulation, representation

Shannon's works on Boolean algebra and information theory are the very foundations upon which all modern computations and communications are based (Shannon, 1940, 1948). Here, we called for article submissions that apply these seminal concepts to information processing in the brain. It is worth accentuating the status quo to provide some pertinent context: “*We don't know how the brain stores anything, let alone words*” (Poeppel and Idsardi, 2022). In addition, there are currently two major competing theories: Hebbian synaptic hypothesis (Mayford et al., 2012; Kandel et al., 2014), and cell-intrinsic hypothesis (Gallistel, 2017, 2021; Gold and Glanzman, 2021; Akhlaghpour, 2022). For an introductory overview, please see Tee and Taylor (2022).

Five articles were accepted and published as part of this Research Topic. Pio-Lopez et al. presented results from four computer simulations and one experimental test to establish an information processing connection between cell biology (i.e., morphogenesis) and neuroscience (i.e., active inference). Gallistel et al. presented a Bayesian algorithm and three experiments on firing pauses of cerebellar Purkinje cells associated with eyeblink conditioning, in support of the cell-intrinsic hypothesis. Ricci et al. presented results from six computer simulations of the cerebellar Purkinje cell using a leaky integrate-and-fire neuron to demonstrate the computational feasibility of the cell-intrinsic hypothesis. Fitch elaborated on the hypothesis that information in the brain is stored not only in the synapse and inside each cell, but also in the dendritic structure and cell-to-cell connectivity. Madani presented the performance of a novel model for learning hierarchical concepts based on an information theoretic score, in comparison with the performance achieved by synaptic-based artificial neural network models and n-gram language models.

While significant progress has been made in the computational aspect of the brain, the communications aspect remains very much unresolved. For example, all communications systems require a modulation scheme to carry the information from its point of origin to a given destination (e.g., information storage and retrieval). What modulation scheme is employed in the brain? For a more detailed examination of the modulation problem, please see Tee and Taylor (2020). To date, the most plausible modulation scheme that falls consistently within Shannon's framework is *Interspike Interval* (ISI), known in communications literature as *Differential Pulse Position Modulation* (DPPM) (Berger and Levy, 2010; Tee and Taylor, 2020). What we still do not know is the alphabet size (i.e., the number of distinct symbols) characterizing such a DPPM scheme. Equivalently, what we still do not know is how many bits of information each modulation symbol represents (or conveys). Previous works have concluded that the brain may be computationally representing subjective probabilities with four bits of precision (Tee and Taylor, 2019) and subjective values with five bits of precision (Tee and Taylor, 2021a). How might such computational representation precisions translate into modulational representation precisions? The open-access Timing Database (Aydogan et al., 2023), consisting of 68 datasets from eight different tasks, may be a helpful resource for a deeper exploration into these representation questions.

One common assumption in computational neuroscience is that the brain operates at Shannon's channel capacity (Berger and Levy, 2010; Levy and Calvert, 2021). In communications systems, transmitting a message at a rate arbitrarily close to capacity is theoretically possible (as per Shannon's seminal work). However, in practice, operating at (or near) capacity is not necessarily feasible, partly because this requires the use of advanced *Error Correction Codes* (ECCs), that transform the message in a longer codeword. For example, in Chung et al. (2001), a *Low-Density Parity-Check* (LDPC) ECC is shown to closely approach Shannon's capacity limit by using a codeword length equal to 10^7^ bits (i.e., 10 million bits). Such a length requires big data buffers (i.e., memory) to temporarily store the entire message during encoding and the received codeword at the decoder, which can present a storage issue/challenge. Furthermore, the entire codeword needs to be received before the decoding process can begin, resulting in a time delay. For instance, a block of 10^7^ bits transmitted at 1 Mbps takes 10 s to be delivered; even if the transmission rate were 10 Mbps, it would still take 1 s to arrive. Finally, the decoding process may not be computationally trivial and certainly introduces an additional time delay. All these constraints can make error correction encoding and decoding unfeasible to operate near Shannon's capacity if the decoding buffer requirement and time delay are unacceptable. In contrast, *Random Access Memory* (RAM) in modern computers employs much simpler and shorter ECC, without operating near Shannon's capacity. For example, DDR5 SDRAM employs a codeword length of 136 bits (i.e., 128 data bits and eight ECC parity bits generated using a Hamming code; Rooney and Koyle, 2019), enabling the decoder to operate with a very small buffer and negligible time delays. Given that advanced ECC is needed to operate near Shannon's capacity, the constraints related to the size of the decoding buffer and the maximum time delay make it rather unlikely for the brain to achieve these conditions. Consequently, the common assumption that the brain operates at Shannon channel capacity may need to be revisited. It is also worth noting that the simplest possible ECC that does not require a buffer and has the lowest possible time delay is a *repetition code*. Such a scheme is, therefore, neurobiologically plausible, especially for real-time processes in the brain, such as visual and auditory systems (Tee and Taylor, 2021b).

We want to thank all authors for their submissions to this Research Topic, and the reviewers for evaluating the submissions. We hope that this Research Topic will provoke some insights and ideas for future research on information processing in the brain. Lastly, we dedicate this Research Topic to the late Desmond P. Taylor (IEEE Communications Society, 2022; Royal Society of New Zealand Te Apārangi, 2022; Zuckerman, 2022).

## Author contributions

JT: Writing—original draft, Writing—review & editing. GV: Writing—original draft, Writing—review & editing.
